# Complicated Colonic Diverticulitis Presenting as Vaginal Bleeding: An Unusual Presentation

**DOI:** 10.7759/cureus.10595

**Published:** 2020-09-22

**Authors:** Adnan Zafar, Thanuja Neerukonda, Yousaf Zafar

**Affiliations:** 1 Internal Medicine, CMH Lahore Medical College and Institute of Dentistry, Lahore, PAK; 2 Internal Medicine, Brandon Regional Hospital, Brandon, USA; 3 Internal Medicine, Naples Community Healthcare, Naples, USA

**Keywords:** divirticulitis, vaginal discharge

## Abstract

Colonic diverticula are small outpouchings from the colonic lumen secondary to mucosal herniation through the colonic wall. Clinical presentation usually involves an acute or subacute onset of abdominal pain typically localized in the left lower abdominal quadrant as well as nausea, low-grade fever, bowel habit changes, and elevated inflammatory markers. We present a case of complicated colonic diverticulitis with vaginal fistula with eventual formation of abdominal abscess, presenting as vaginal bleeding and pelvic pain.

## Introduction

Colonic diverticula are small outpouchings from the colonic lumen secondary to mucosal herniation through the colonic wall [1]. Diverticulitis is characterized by inflammation of one or more adjacent diverticula and the surrounding colon. Diverticulosis is a necessary indication for the development of diverticulitis. An estimated 15%-20% of individuals with diverticulosis coli will develop acute complicated diverticulitis in their lifetime [2]. Increasing age is an important risk factor for both diverticulosis and diverticulitis as well as obesity and smoking [3]. Clinical presentation usually involves acute or subacute onset of abdominal pain typically localized in the left lower abdominal quadrant as well as nausea, low-grade fever, bowel habit changes, and elevated inflammatory markers [2]. Diverticulitis can be determined as complicated or uncomplicated. Complications include abscesses, peritonitis, obstructions, and/or fistulas [4]. We report a case of a clinically unique presentation of complicated colonic diverticulitis associated with an abdominal phlegmon and vaginal fistula, eventually resulting in an abdominal abscess. 

## Case presentation

A 62-year-old female with a past medical history of type 2 diabetes mellitus, peripheral arterial disease, hypertension, gastroesophageal reflux disease, and hypothyroidism presented with a two-day history of vaginal bleeding and lower pelvic pain. At the time of admission, her temperature was 98.4 degrees Fahrenheit, her heart rate was 86 beats per minute, and her respiratory rate was 21 breaths per minute. Additionally, physical examination showed regular heart rate and rhythm with no murmur; breath sounds clear to auscultation with no wheezes or crackles; positive bowel sounds were present and the abdomen was nondistended, soft, and nontender. 

There were no electrolyte disturbances: sodium level of 141 mmol/L, potassium level of 3.9 mmol/L, chloride level of 103 mmol/L, and bicarbonate level of 24 mmol/L. She did present with a leukocytosis of 16.57 10*3/cmm and a slightly decreased hemoglobin level of 10.0 g/dL. In the ED, ultrasound of the abdomen and pelvis revealed a 6.9 cm x 5.9 cm x 5.6 cm indistinct pelvic mass involving the sigmoid colon, cecum, and terminal ileum. Ultrasound also showed diverticulosis of the sigmoid colon. During admission, she underwent a colonoscopy that showed no luminal mass but only diverticulosis. Colon barium enema demonstrated sigmoid colon diverticulosis with a connection between the colon and pelvic mass. Therefore, general surgery was consulted and she underwent exploratory laparotomy with biopsy of the indistinct pelvic mass and diverting loop ileostomy. Biopsies at this time did not find any malignant cells but found benign fibro-adipose and smooth muscle admixed with mucus, blood, and a small fragment of colonic mucosa with chronic inflammation.

The differential diagnosis for this case presentation included malignant neoplasm, infectious process, and complications secondary from perforated diverticula with secondary fistula formation. Malignancy seemed unlikely as systemic symptoms were not present including weight loss or fatigue. At this time, her tumor markers including carbohydrate antigen (CA) 125 protein, CA 19-9 protein, and carcinoembryonic antigen (CEA) were also checked and were negative. Diagnosis at this time was thought to be perforated diverticulitis with phlegmon and subsequent fistulization into the vagina. 

**Figure 1:**
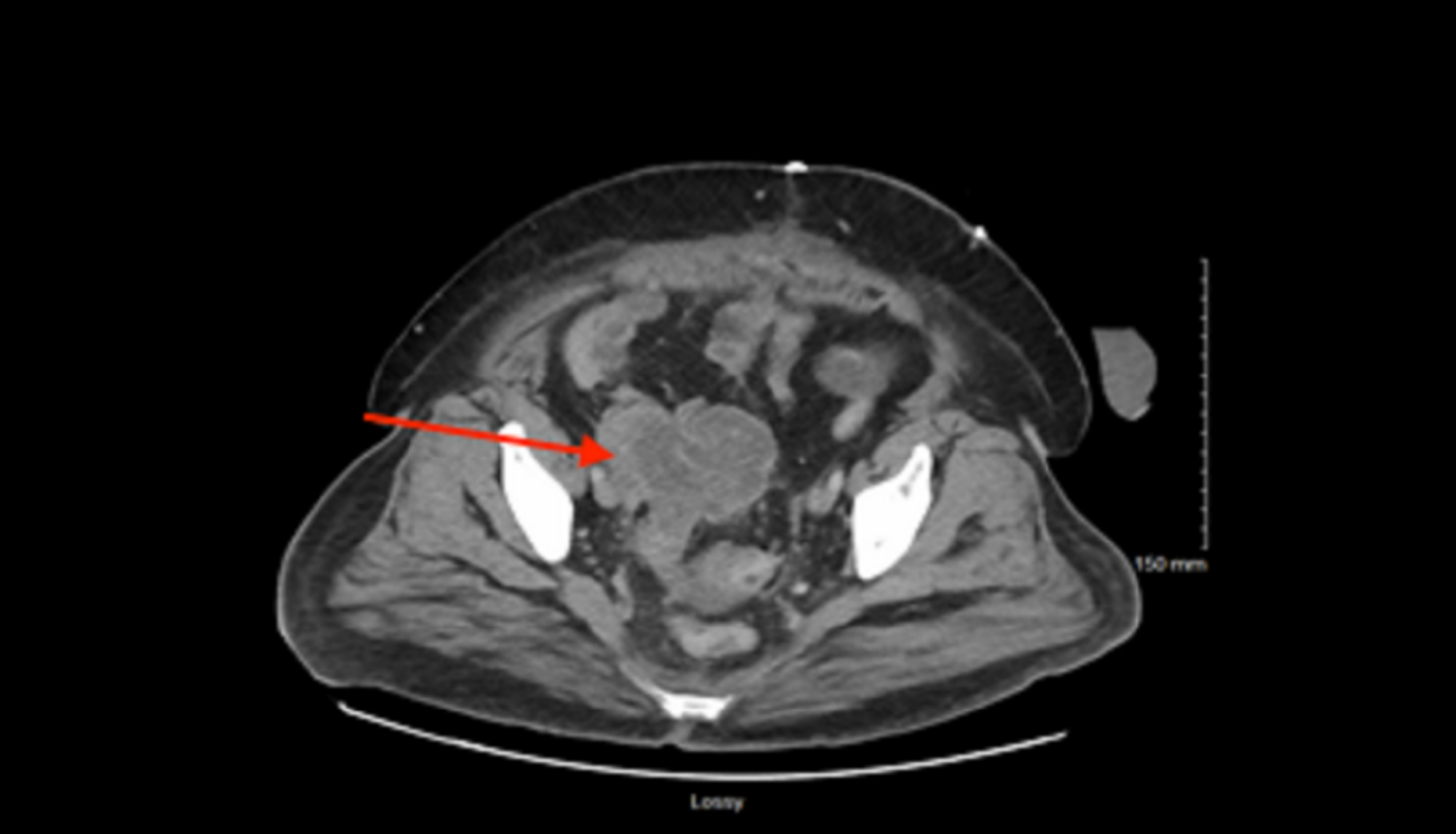
CT of the abdomen and pelvis showing the mass.

Throughout her admission, IV metronidazole 500 mg every eight hours and cefepime 1 mg every 12 hours were begun. However, she was eventually switched to IV vancomycin and ertapenem seven days after admission because CT of the abdomen and pelvis demonstrated a new, small peripheral lenticular shaped fluid collection anteroinferior to the original pelvic mass, concerning for a possible abscess (Figure 1). During her exploratory laparotomy, the perforation was difficult to resect and strongly fixed; thus, it was not removed. She continued to show improvement with IV antibiotics throughout admission; her pain declined and she had no more episodes of vaginal bleeding. Eventually, she was switched to oral antibiotics and discharged home with a 10-day course of ciprofloxacin 500 mg twice daily and amoxicillin-clavulanic acid 875 mg twice daily.

By the time of discharge, the patient was status postexploratory laparotomy with intact, clean, and dry ileostomy in the left lower abdominal quadrant with properly draining liquid stools in the ileostomy bag. Her clinical symptoms resolved including pelvic pain, abdominal pain, and vaginal bleeding. She was discharged home with a 10-day course of ciprofloxacin 500 mg twice daily and amoxicillin-clavulanic acid 875 mg twice daily. 

## Discussion

The incidence of diverticulosis and diverticulitis has progressively increased in Western countries. Some 80%-85% of patients with diverticulosis are asymptomatic and 15%-20% present with symptoms. Symptoms vary on the extent of the diverticular disease [5]. However, symptoms commonly present with acute or subacute onset of abdominal pain typically localized in the left lower abdominal quadrant as well as nausea, low-grade fever, bowel habit changes, and elevated inflammatory markers. Furthermore, diverticular disease can be considered as uncomplicated or complicated disease. Uncomplicated diverticulitis involves thickening of the colon wall and peri-colonic inflammatory changes while complicated diverticulitis is seen in 12% of patients with diverticulitis [2]. Complicated diverticulitis includes the presence of abscess formation, peritonitis, obstruction, and/or fistula [4].

 Fistulas occur in 2% of patients with complicated diverticular disease. Usually fistulas can involve any organ in the pelvis including vaginas, uterus, fallopian tubes, perineum, ureter, and other parts of the colon [6]. Additionally, simultaneous complications are common as seen in our report with an abdominal phlegmon, vaginal fistula, and further progression of abdominal abscess [7]. Reports have identified diverticular disease fistulas but never with symptoms of vaginal bleeding and pelvic pain. Our literature search revealed one case report that detected a sigmoido-cecal fistula in the setting of acute sigmoid diverticulitis in an individual presenting with left lower quadrant abdominal pain [8]. Furthermore, one study looked at a series of genital fistulas that resulted from diverticulitis. They identified 13 cases: 10 fistulas involved the vagina, one the vagina and the bladder, one the fallopian tube, and one the uterus. All but one presented with malodorous vaginal discharge. Interesting enough, all patients with vaginal lesions had previously undergone total hysterectomy [9]. Our case report explicits a patient with a vaginal fistula who has not undergone previous total hysterectomy and instead presented with vaginal bleeding rather than maladorous vaginal discharge. Additionally, she had an associated abdominal abscess. Another report did identify a female presenting with pelvic pain and vaginal spotting; however, she had a tuboovarian abscess due to colonic diverticulitis [10]. Controversially, our patient presented with an abdominal abscess and vaginal fistula rather than a tuboovarian abscess.

 The aim of this case report was to acknowledge that even though diverticular disease is well studied already, it may present in an unusual and unique way. Complications of diverticular disease have all been represented in literature. Genital fistulas have also been reported in literature in addition to combinations of complications. However, clinical presentation of vaginal bleeding associated with an abdominal abscess and vaginal fistula has not been reported in literature so far. Interestingly, we came across this unique presentation and this report advances the idea that colonic diverticulitis complications can manifest unusually. In a patient with a history of diverticulitis presenting with any sort of unusual presentation, diverticular disease complications should be considered.

## Conclusions

This case demonstrates an important clinical complication of colonic diverticulitis, particularly abdominal phlegmon, vaginal fistula, and further abdominal abscess. Colonic diverticulitis complications have been extensively identified in scientific literature; however, this presentation is unusual and suggests that complications of diverticulitis can manifest in many different unique clinical presentations such as in our report of vaginal bleeding. Surgical intervention and broad-spectrum antibiotics can sufficiently treat patients who may develop fistulas from the colon to adjacent organs.
